# Supplementary material for: SampleQC: robust multivariate, multi-cell type, multi-sample quality control for single-cell data
Source: Genome Biol. 2023 Feb 10;24:23. doi: 10.1186/s13059-023-02859-3 (PMC9912498; doi:10.1186/s13059-023-02859-3)

Additional File 1: Fig. S1 Biaxial empirical density plots of QC metrics from selected samples and datasets. Colour indicates concentration of cells with the same QC metrics. *log_counts* indicates `log10` of library sizes, *log_feats* indicates `log10` of number of detected genes, *logit_mito* indicates `qlogis` i.e. inverse logistic function of mitochondrial proportion. For list of datasets, see Table .

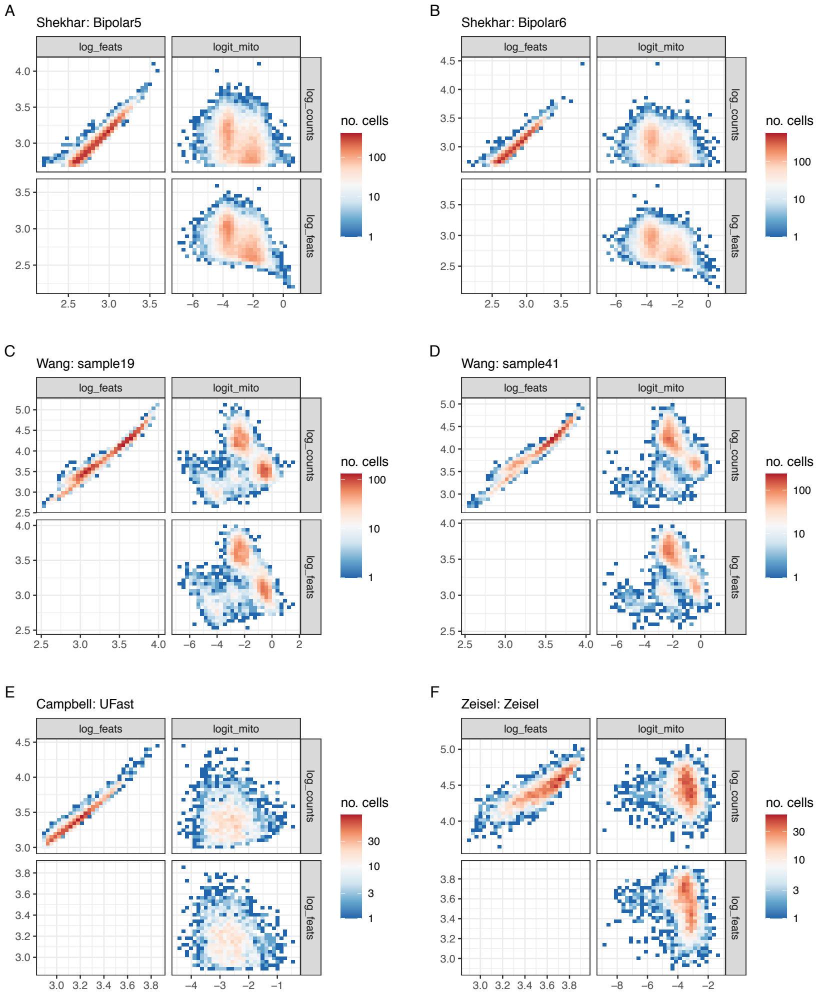

Additional File 1: Fig. S2 QQ plots of QC metrics from selected samples and datasets. y-axis is theoretical quantiles of normal distribution, using mean and standard deviation estimated from transformed QC metrics. Median of QC metric used to estimate mean, MAD of QC metric for standard deviation. x-axis is observed values of selected QC metrics in each sample. *counts_k* indicates library sizes in thousands, *feats_k* indicates number of detected genes in thousands, *mito_pct* indicates percent of mitochondrial reads, *log_counts* indicates `log10` of total reads per cell, *log_feats* indicates `log10` of number of detected genes, *logit_mito* indicates `qlogis` i.e. inverse logistic function of mitochondrial proportion, *log_mito_prop* indicates `log10` of proportion of mitochondrial reads. For list of datasets, see Table .

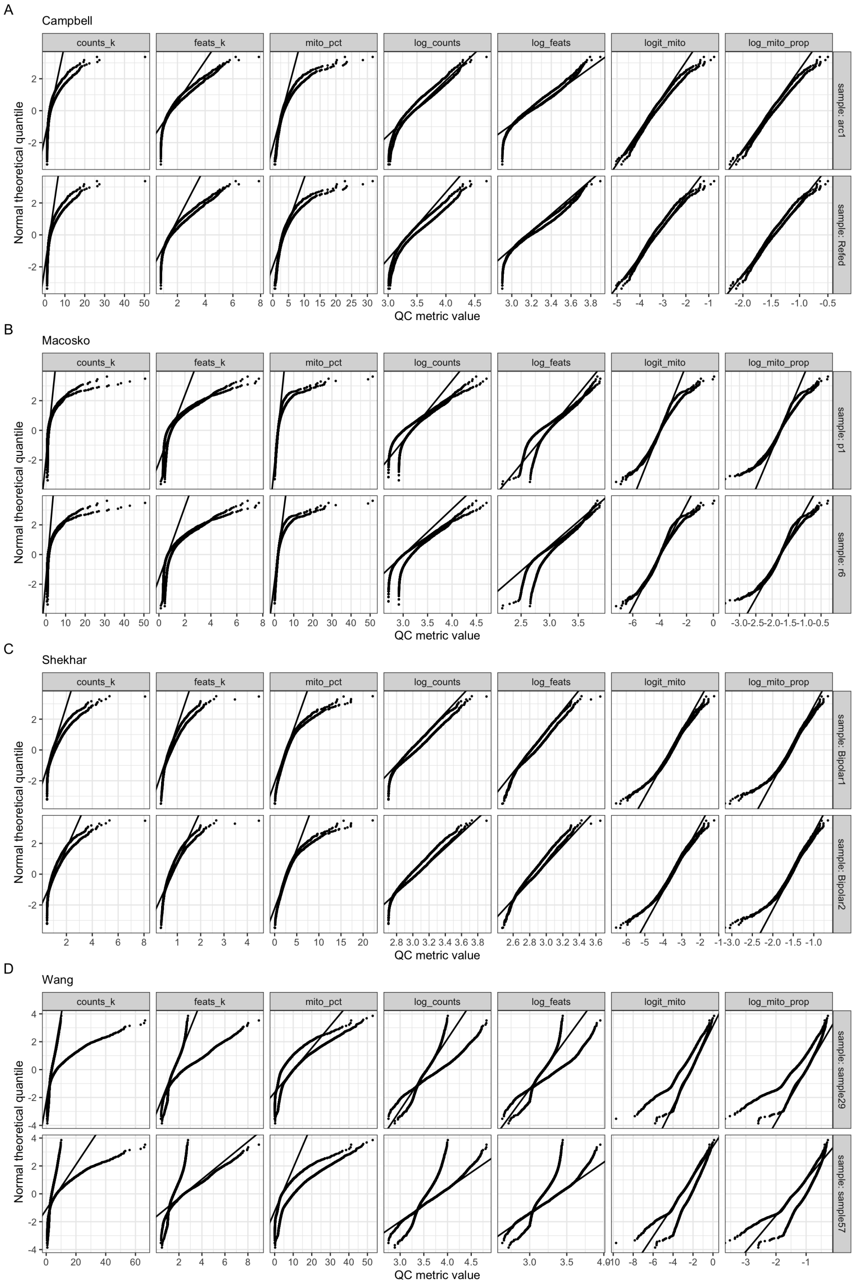

Additional File 1: Fig. S3 QC method comparisons on unimodal simulated data. **A** True simulated outliers (red). **B** Multivariate density plot of simulated data, showing `SampleQC` fits. **C** Mahalanobis distance to nearest cluster under fitted `SampleQC` model. **D**, **E**, **F** Outliers detected by `SampleQC`, `scater` and `miQC` respectively.

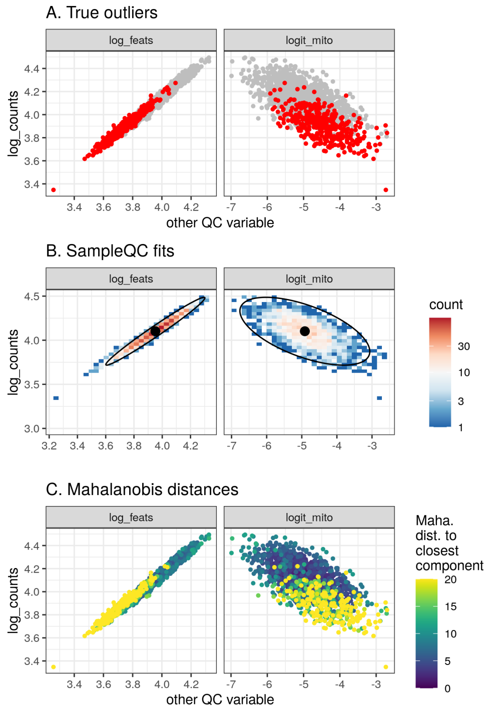

Additional File 1: Fig. S4 PR curves for individual samples. PR curves for 20 randomly selected samples from simulated experiments. Precision is proportion of cells reported as 'good' that are actually 'good'; recall is proportion of all 'good' cells that are reported as 'good'. Samples in first row are unimodal, i.e. they are composed of one celltype; other samples have either 2 or 3 celltypes. Dashed lines show results for simulations where QC celltypes have QC metrics modelled as multivariate t-distributions with 5 degrees of freedom, and other simulation parameters were kept constant.

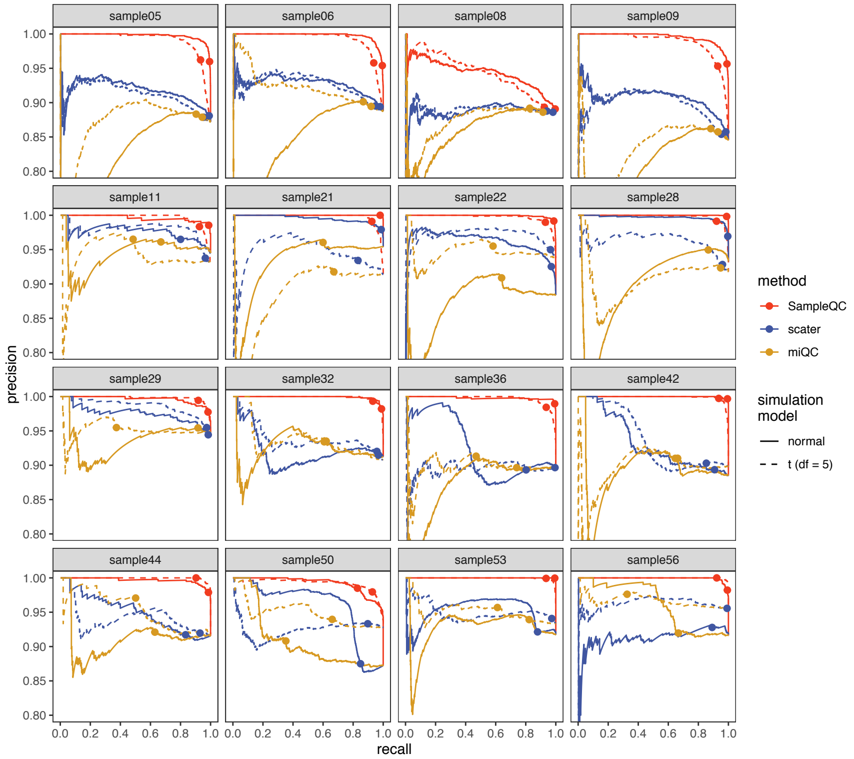

Additional File 1: Fig. S5 Precision-recall curves split by number of celltypes present. Modality indicates number of modes present in the distribution, i.e. the number of celltypes.

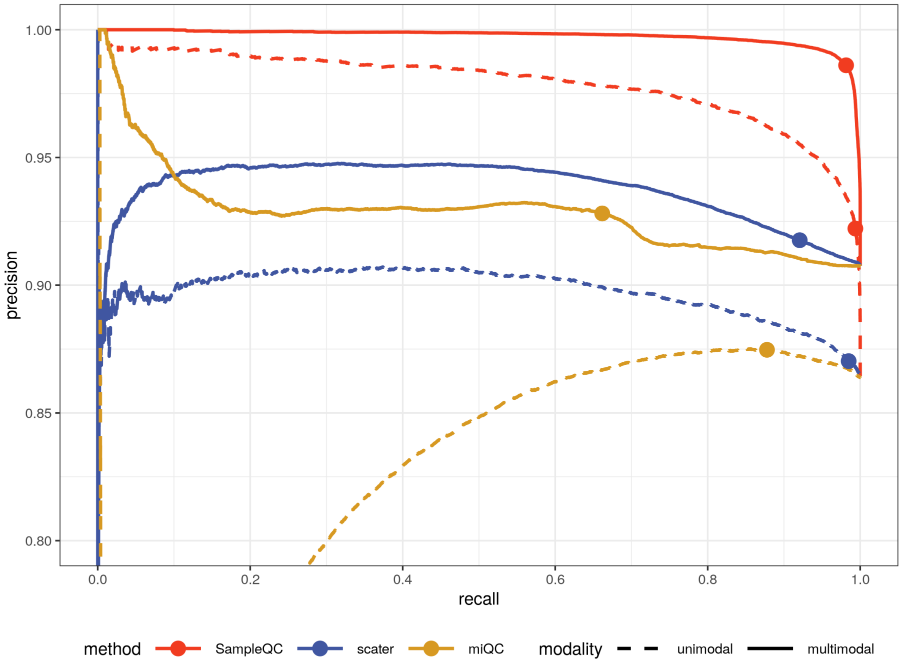

Additional File 1: Fig. S6 Split of misreported good cells by celltype.

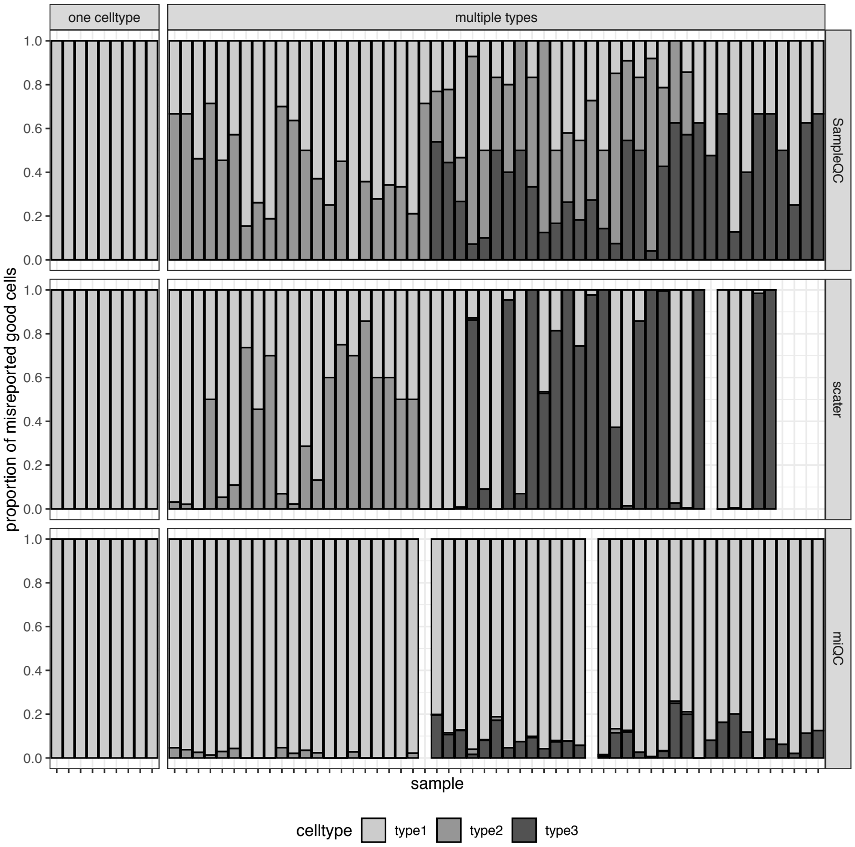

Additional File 1: Fig. S7 Robustness to choice of k. **A** `SampleQC` fits to simulated data, for combinations of `k_fit` and `k_sim`; columns are `k_fit`, rows are `k_sim`. Each fit illustrated by randomly selected sample for each combination. Note that for some combinations of `k_fit` and `k_sim`, `SampleQC` was not able to fit the data; these are blank. **B** Precision-recall curves of identification of 'good' cells. Solid lines show curve where `k_fit` = `k_sim`. Dots show default cutoff ($< 1\%$ Chi-squared tail).

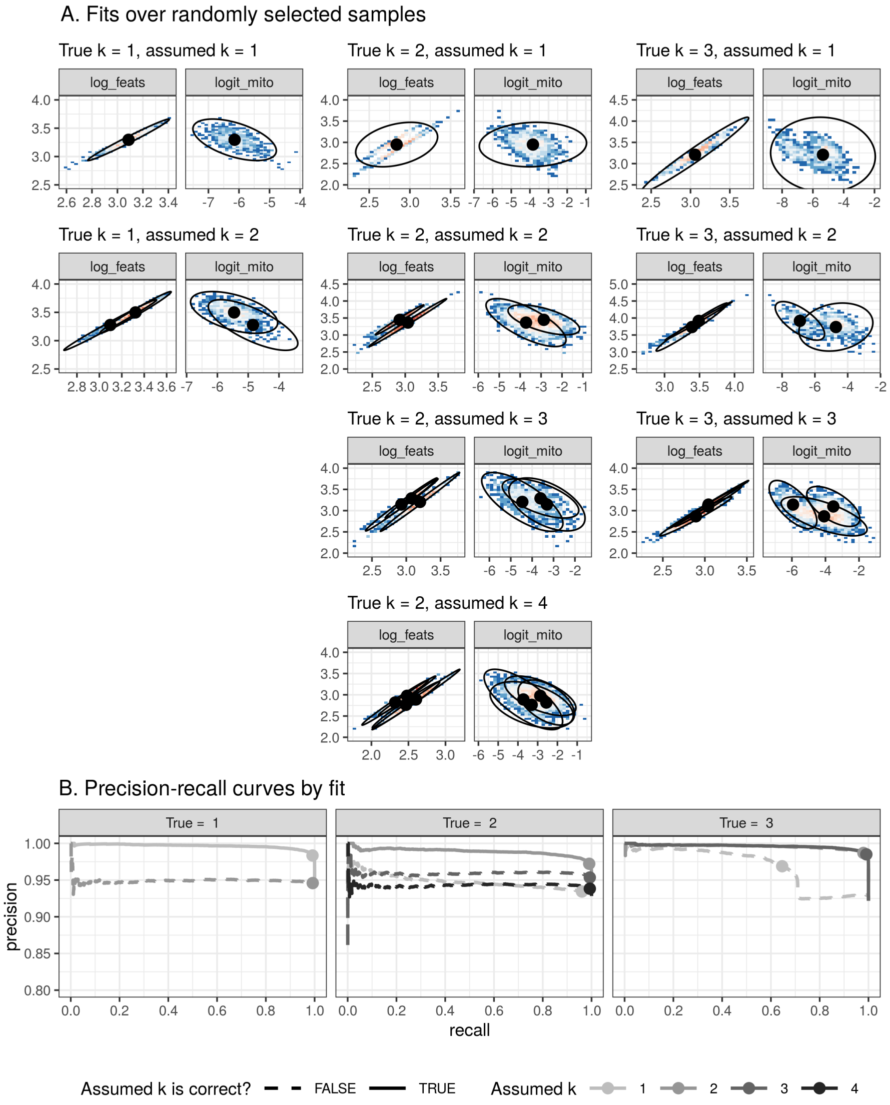

Additional File 1: Fig. S8 Sample-to-sample distance embedding (MDS). **A** Sample group, as derived from `SampleQC` clustering **B** Sequencing batch **C** Mitochondrial proportion (categorized) **D** Number of cells (categorized). **E** Median counts per cell **F** Median log2 splice ratio per cell.

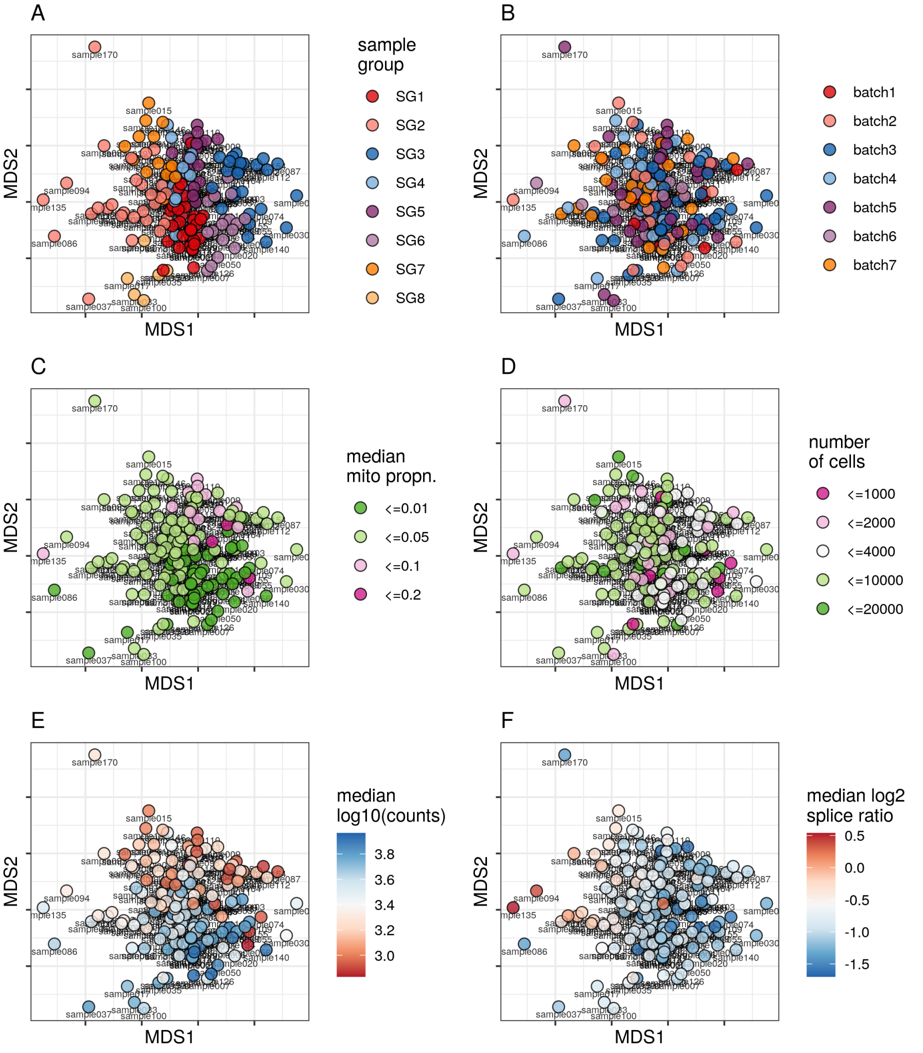

Additional File 1: Fig. S9 Comparison of Mahalanobis distances under `SampleQC` model and distances under expected Chi-squared distribution. Mahalanobis distances for Roche data and model fit in Figure 5, samples selected at random.

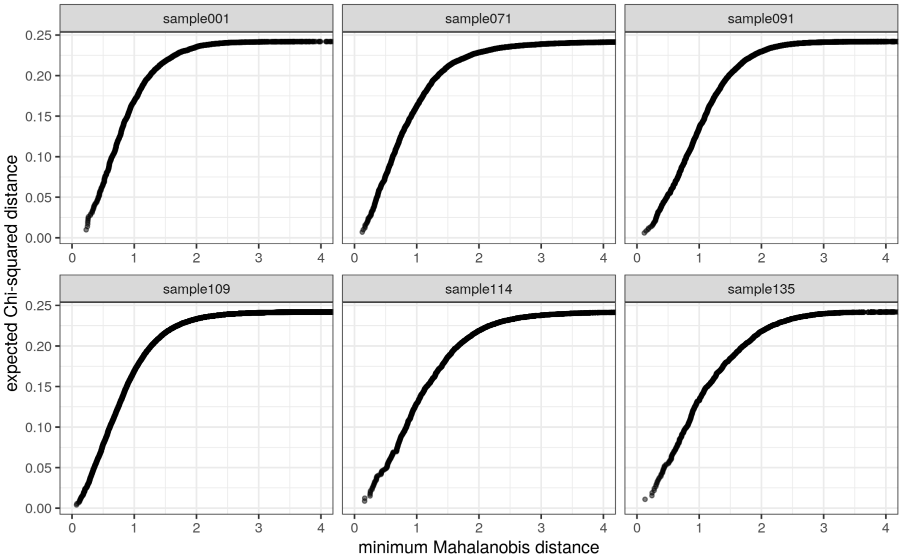

Additional File 1: Fig. S10 `SampleQC` outputs for selected CITE-seq sample. Biaxial distributions of CITE-seq QC metrics for PBMCs [25], annotated with fitted models fitted by `SampleQC`. Selected sample *H1B2ln2* from batch 2; distributions and fits for other samples are similar.

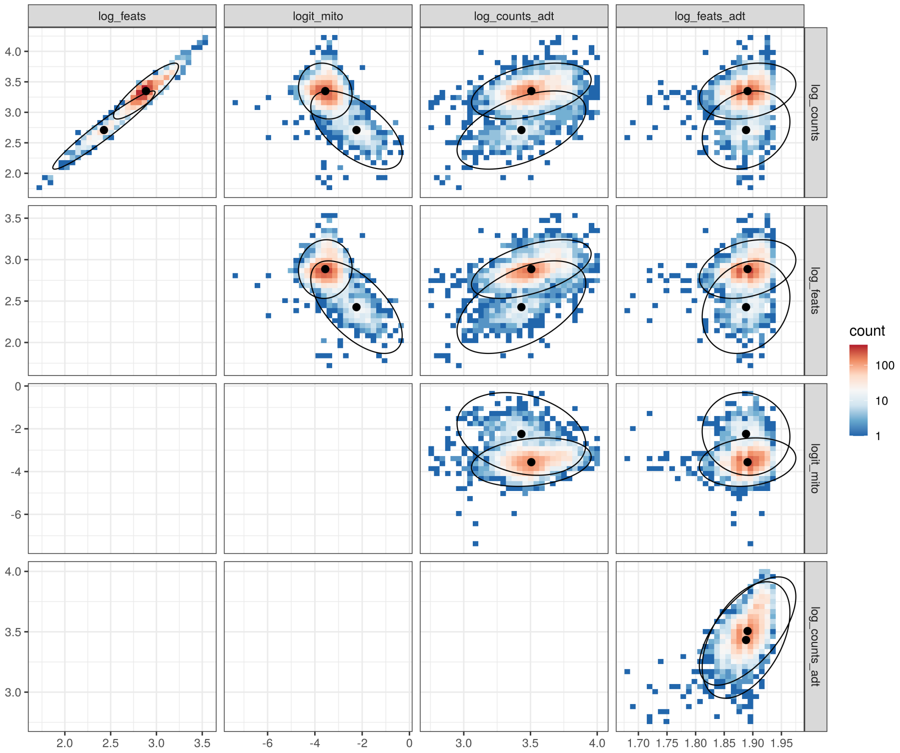

Additional File 1: Fig. S11 Identifying outlier cells via standard single cell clustering. Selected sample is ovarian cancer tissue, *16030X4* from the HGSOC dataset (see section). **A** UMAP applied to highly variable genes, coloured by Louvain clustering [16]. **B**-**D** QC metrics for each cell, truncated to median + / - 2 MADs. **E** QC metric distributions of clusters in **A**.

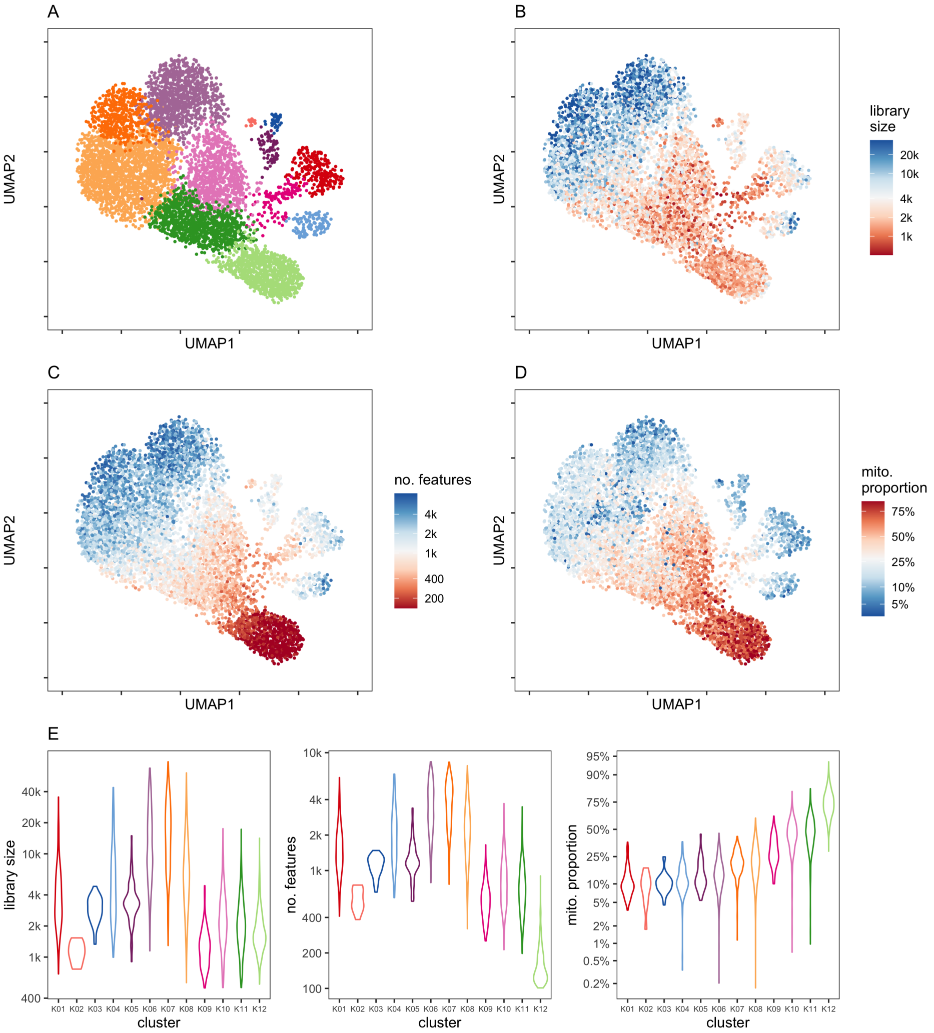

Supplement: Supplementary file 1 — Additional file 1. Supplementary Figures. [file 13059_2023_2859_MOESM1_ESM.pdf]
