# Supplementary material for: SampleQC: robust multivariate, multi-cell type, multi-sample quality control for single-cell data
Source: Genome Biol. 2023 Feb 10;24:23. doi: 10.1186/s13059-023-02859-3 (PMC9912498; doi:10.1186/s13059-023-02859-3)
Supplement: Supplementary file 3 — Additional file 3. Authors’ response to first round of review. [file 13059_2023_2859_MOESM3_ESM.pdf]

In what follows below, the original text from the reviewers is in *grey italic*, while our responses are in black straight text.

Editorial comments

As you will see from the reports, both referees are broadly favorable and find the work of potential interest, but they raise important issues that we must ask you to address, in the form of a revised manuscript, before we reach a final decision on publication. In particular, it seems to us to be essential that Referee 1's concerns about cells classified as "poor quality" are resolved. Referees 2 and 3 both have concerns relating to outliers and how they are treated and discussed. Please ensure that these and all other issues raised by the referees are addressed in full.

We thank the editors for this assessment. In our revision, we have focused on the point about what cells are deemed poor quality and provided further analyses and descriptions.

We are considering your manuscript as a 'Method' article. This is our format for publishing articles that describe a methodological innovation that is a significant advance over published methods and likely to be of broad utility, but that do not provide significant biological insights. When revising the manuscript, please ensure the manuscript conforms to our style for Methods articles (see https://genomebiology.biomedcentral.com/submission-guidelines/preparing-your-manuscript/method); specifically, the abstract should be under 100 words. Please note that if we decide to publish your manuscript we will require that the source code is made publicly available under an open source license compliant with Open Source Initiative, with the license clearly stated in the manuscript. The source code should be deposited in a public repository, such as for instance github, with the accession links included in the manuscript. We also ask that the version of source code used in the manuscript is deposited in a DOI-assigning repository, such as zenodo, with the link also included. All this information should be listed in a separate Availability of Data and Materials section of the manuscript.

We have double checked now that our manuscript conforms to the Method article guidelines. In particular, our abstract is now 100 words, the source code (both the software and analyses) is available from a Zenodo DOI and the relevant information is documented in the 'Availability of Data and Materials' section.

Reviewer #1: Review

Macnair & Robinson propose a version of a Gaussian mixture model to be applied to summarized QC metrics in scRNA-seq data to identify outliers corresponding to 'bad' cells.

The model, which uses robust inference to fit in the presence of outliers as well as including batch-specific mean shifts, is beautifully designed with a very nice corresponding R package that could find use in many fields beyond scRNA-seq.

The use of logistic transformation of the mitochondrial proportion is a great idea; the logistic transformation of fractions is very underutilized in the field.

We thank the reviewer for this positive assessment; indeed, the logistic transformation is an underutilized trick.

The simulation strategy outlined by authors is comprehensive and appropriate. Data is simulated according to the specification of the model proposed by the authors. They demonstrate clearly that the model will successfully be able to recover the simulation conditions better than alternatives. While the results are positive and encouraging (mixture models are hard to get fitting correctly even for data simulated directly from the models!), it only demonstrates inference is possible rather than truly identifying 'bad' cells.

Indeed, the reviewer highlights our exact challenge in this work. Our simulation encapsulates previous suspicions about ‘bad’ cells, but indeed we do not have concrete information about what ‘bad’ cells really are and how they manifest. We have added a statement about this in the Discussion: “Ultimately, our simulation highlights that inference from our model is possible, but does not directly show that cells are `bad'.”

As opposed to many modeling + single cell papers, here the authors have properly quantified the performance of theirs and alternative metrics with precision-recall curves. It would be great if these results were presented in Figure 2, which is more useful than the example scatter plots currently presented which doesn't quantify the performance and instead relies on mental pattern matching by a reader.

We would be happy to change the progression of figures based on the Editor’s feedback, but ultimately we also wanted to highlight the challenges in visualizing cells deemed low quality, and some of the exploratory analysis that SampleQC offers. We think that Figure 2 highlights nicely the challenge beyond the schematic that is shown in Figure 1.

Cells are labeled as 'poor quality' or 'bad' if they are far into the tails of the Gaussian mixture components. It is not clear why these cells would be of poor quality as opposed to following biological variation with wider distribution than can be represented by a multivariate normal. Often real world data need to be modeled with t-distribution rather than Gaussian distributions to properly mimic the observed data.

This is an interesting point; however, in practice, the cells that are called as outliers are also in the tails of t-distributed data. The robust inference machinery we used can also handle t-distributions that have heavier tails: the estimates of mean and correlation remain accurate,

although the distance from the means at which a cell is called as an outlier will be slightly larger. We have tested SampleQC on t-distributed simulated data, and find that while there is a small negative impact on performance, SampleQC still outperforms the other methods considered (see Supp Fig S4 in revised manuscript).

Relatedly, the assumption of the cell QC GMM is that large clusters correspond to useful cells while outliers correspond to 'bad' cells. However, in practice it is common to find a distinct cluster between QC metrics which is enriched for various markers of cell death or degradation.

The reviewer points to a valid alternative approach to QC. We have added additional analysis (with results shown in Supp Fig S11), where we applied this approach to single cell data from ovarian cancer tissue. We found that while some clusters should clearly be excluded, other clusters showed a wide range of QC metrics, showing that this approach to excluding poor quality cells may be more difficult in samples not comprising healthy cells. We have also observed similar results in snRNA-seq samples from brains with neurodegeneration; it may be that there are substantial differences in QC distributions between model-based (e.g., mouse) and clinical samples. We have added text to the Discussion section on this point.

It is a reasonable theory that different biological cell types will produce different clusters in 'QC space'. But for none of the datasets is it demonstrated that different cell types indeed have different QC distributions. For the sake of a reader it would be appropriate to relate examples of different kinds of cell types that can have different QC values and why.

Overall, our entire approach is motivated by real datasets, as shown in Figure 2 and Supplementary Figure 1, where we “see” directly different mixtures in ‘QC space’ after making standard assessments of cells called (e.g., by CellRanger). However, this does not show directly how cell types are distributed. A reference for this phenomena is Germain et al. 2020, Figure 3 (screenshot shown at the right); this is also explored at the tissue level in Osorio et al. 2020.

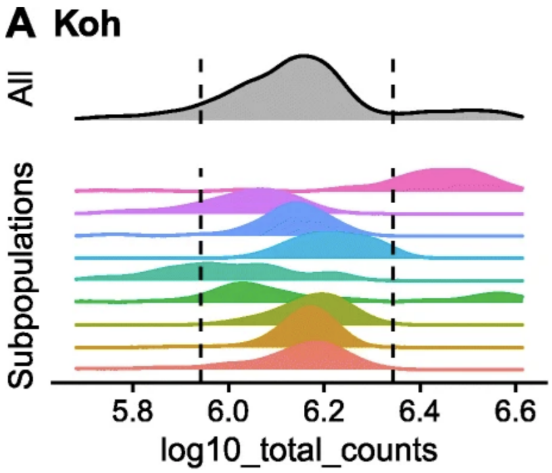

One additional caveat here is that in most datasets, annotation takes place after filtering, so we do not always have the option to know if filtered cells could in fact be annotated. That is, we are lacking real datasets that have good/bad status as well as celltypes annotated.

Another assumption is that the majority of cells will be 'good'/useful. However, for some experimental systems with particularly fragile cells a majority will be 'bad'/dead. Since modern platforms can capture tens of thousands of cells per sample it tends to not be an issue since you can still capture hundreds of useful cells. The authors should provide guidance regarding these settings.

This is an interesting point. It is worth highlighting here that SampleQC allows the flexibility to specify certain clusters for exclusion if it is known in advance whether some subsets are suspecting to be low quality. We have added comments in the user guide about these issues.

The authors write that excluding cells with more than 5%/10% mitochondrial reads is a common approach to QC. It would be good if the authors demonstrated with a few recent example publications to illustrate the prevalence of this practice. The authors do have a reference to a review recommending this, but this does not indicate to a reader that this is a widely used practice in the field.

The reality is that there are many practices used in the field (i.e., no standard) and so we have rephrased the comment to now mention that filtering based on mitochondrial percentage is recommended (e.g., in a "Best practices" article and in the OSCA book) and that 5%/10% is a specific example that was previously proposed.

It is stated that the current industry standards for QC are 'scater' and 'Seurat'. It would be good to provide examples of the use of this strategy. Reading papers from the field, it seems far more common to make arbitrary cutoffs on a couple of custom metrics in each paper.

We based this comment about "industry standards" according to what recommendations people find when they search for single cell pipelines, including the OSCA (Orchestrating Single Cell Analysis) online book and Seurat vignettes. As mentioned above, the reality is that data analysts implement various (presumably sensible but arguably arbitrary) strategies. We have reworded the text to convey this sentiment.

The authors mentions that applying QC filtering to each sample individually introduces bias, but this is not investigated or presented in this paper.

Towards the end of the paper the authors state that jointly analyzing all samples increases sensitivity by borrowing strength across samples. There is however no discussion of comparison of performance of analyzing the samples individually as opposed to jointly.

We consider that "applying QC filtering to each sample" is represented by 'scater', since estimates are based on MADs calculated separately for each sample. There is no way to test SampleQC on single samples, because it explicitly requires multiple samples to estimate the mixture of Gaussians.

A large issue is how this method fits into an analysis workflow. The central claim is that different cell types of useful cells will produce different densities of QC metrics. For a practitioner to make use of data with multiple cell types, these need to be annotated for further analysis. But in this setting 'bad' cells would form their own clusters or fail to be annotated in more supervised settings. Since the work of clustering cells and labeling still needs to be done, it would seem easier to identify 'bad' cells at that step.

In our experience, we have observed that both models - clusters of bad cells, and continua of celltypes with decreasing quality - occur in practice. As discussed above (see response to reviewer comment beginning "Relatedly, the assumption of the cell QC GMM"), we have added new analysis that shows that excluding clusters of bad cells is not appropriate for all datasets.

Ultimately, at least for clustering, the user needs to draw a line at some point and the results (e.g., that are already based on sets of highly variable genes) are dependent on those choices. We have added Discussion about this in the paragraph starting “QC does not need to be conducted entirely at the filtering stage”.

It would be appropriate to discuss which QC metrics to use, and how they relate to potential failure modes in the scRNA-seq process. The authors do discuss this for the intronic vs exonic ratio of reads for snRNA-seq and offer a plausible theory (albeit speculative, which could use a qualifier to indicate this).

We agree that this would be helpful to users, and have added a paragraph in the Discussion section.

Upon seeing the facet_grid in Figure S2 it is not obvious what the vertical axis represent. It appears to be individual samples; it would be good if the authors used the 'labeller = "label_both"' option to make this clear.

Thank you for this comment. We have modified the visualization in Figure S2 to better convey the vertical axis.

In summary, the largest issues are: 1) It is not clear whether cells labelled as 'bad' here are 'bad' as opposed to one of the QC clusters being 'bad'. 2) Whether performing this labeling would be useful in the first case since in the clustering step of analysis researchers would identify bad cells anyway.

These are important points. To address them, we have added new analysis of ovarian cancer data (as discussed above), and amplified the relevant parts of the Discussion. Our observations, from ovarian cancer and brain tissue snRNA-seq samples, is that more challenging samples may be less likely to contain one ‘bad’ cluster, and that many cell types / clusters may contain a proportion of cells of low quality. Rather than identifying one ‘bad’ cluster, SampleQC identifies ‘bad’ cells according to being in the low-density regions of the multi-sample-based multivariate QC space. We do not have specific reasons that they are truly low-quality, beyond the observation that they by definition have more extreme QC metrics.

Reviewer #2: ===

Macnair and colleagues develop SampleQC for single-cell RNA-seq data quality control (QC). They do not propose new QC criteria, but an approach to combine multiple existing QC criteria to better identify poor quality cells. To this end, they propose a multivariate Gaussian mixture model, and treat outliers from the fitted model as low quality data points. They evaluate their method using simulated data, which is based on their proposed model, then apply their model to analyze a publicly available real single nuclei RNAseq dataset from human brain.

The proposed method is incremental compared to existing approaches. There are a few merits of the proposed model. Perhaps the main advantage of this approach is that it does not require a predefined threshold for certain QC metrics, such as UMI counts and mitochondria fractions, but the model requires additional parameters that are not easily identifiable from data, e.g. QC celltype numbers and threshold for outliers. The model is not well validated. In addition, there are a number of significant weaknesses as detailed below.

Major comments:

1. The proposed method lacks rigorous validation. Quantitative evaluation is done only for a specific simulated dataset, which is generated based on the assumption their statistical model is correct. This is clearly a circular argument, and the relevance for real data application is unclear. There are a number of published tools for single-cell RNAseq simulations. The authors should consider one of multiple of these tools in order to obtain a more objective evaluation of their method. Also, simulated data has important intrinsic limitations which need to be recognized. To avoid undue optimism in real applications, the authors should also carefully evaluate the performance of their method based on real data. I recognize it can be difficult to determine true error rates for real data, but alternative strategies can be used such as robustness and consistency with literature findings, as commonly done in similar studies.

This reviewer highlights one of our biggest challenges in this work. Despite there being lots of scRNA-seq simulators out there (we even have a study comparing them[1]), we were not able to find one that simulates 'bad'/'low-quality' cells. So, that is why we implemented one from scratch using what little we know about how 'bad' cells manifest, and we believe we are fairly honest about the limitations of simulation (See paragraph in Discussion starting "*It is worth noting that our performance results depend on the choices made in implementing the simulation ..*"). As mentioned above, there is also an absence of real datasets that carry a ground truth to do proper assessments. However, we did apply SampleQC to multiple single cell data types (e.g., CITE-seq), thus highlighting its flexibility. We've expanded on all these aspects in the Discussion (see paragraph starting "*One of the main challenges in this work is the lack of ground truth*") to make the challenges clear.

We do not fully understand the comment "alternative strategies can be used such as robustness and consistency with literature findings, as commonly done in similar studies", because other (newly-proposed) QC tools (e.g., miQC and the original proposals from scran and scater) also

[1] https://doi.org/10.1101/2021.11.15.468676

do not have comprehensive benchmarks, again because there is a lack of ground truth. We have mentioned these limitations quite a bit in the text.

2. A key challenge in QC analysis is to distinguish good from bad data points while considering the underlying biological variability. As noted by the authors, variation in metrics such as UMI counts and mitochondrial fractions may be due to intrinsic biological variation. However, this issue can only be addressed by carefully modeling the sources of biological and technical variations. Instead, a key limitation of sampleQC is that it does not attempt to distinguish biological and technical components. As a result, it is hard to interpret the results. An example is that rare cell types may appear outliers that would be excluded from downstream analysis. Similarly, the term 'QC celltype' is confusing and hard to interpret.

We are not sure that the reviewer's comments here are fully correct. For example, the model does have sample-specific shifts, which handles part of the **technical** variation (e.g., different baseline sequencing depths across samples); and in random sampling, we expect a distribution of sequencing depths (and other QC metrics) for the cells within a single sample, thus representing **biological** variation in the model. Furthermore, the comment about "rare cell types may appear [as] outliers" is exactly why we created our SampleQC model; as long as rare cell types are represented by a 'QC celltype' in one of the samples, it will be retained in the fit of the other samples. We believe that this direct modeling allows SampleQC to be **less** susceptible to filtering rare cell types than any of the competing methods.

Indeed, 'QC celltype' could be a confusing term. However, if you consider the mixtures shown in the motivating examples in Figure 2B, we believe this helps the intuition on the term. We have expanded the caption to highlight the term 'QC celltype' in this context.

3. The benefit of using this proposed QC metric in downstream analyses is unclear. For example, does it help improve the accuracy of cell-type identification, trajectory analysis, etc?

We already know a fair bit about how filtering affects downstream analysis. For example, our work in pipeComp[2] highlighted that "*doublet removal performed worse when combined with lenient or no filtering*" and there is a Section titled "*Excluding more cells is not necessarily better*". Here, it comes back to the comment above regarding the lack of ground truth; we can perform various downstream analyses after various filtering, but in all cases, we do not have annotations for already-filtered cells, so we can only really assess what *more* filtering offers.

We've included this aspect in the commentary in the Discussion, i.e., that we do not have the ground truth in real datasets because filtered cells are not annotated.

4. A key parameter in sampleQC is the number k for GMM. It is unclear how this parameter is chosen. The authors recommend the users to inspect a few plots generated by the program to make their own choice, but this is very difficult without a guiding principle. With this key parameter unsettled, it is also very difficult to evaluate the performance of the model for real data.

[2] https://genomebiology.biomedcentral.com/articles/10.1186/s13059-020-02136-7

Indeed, the number of mixtures is a key parameter in the modeling of mixtures and this has been a well-studied though unsolved problem in statistics for decades. In our experience and as discussed in the User Guide of the SampleQC software, it becomes quite clear in the analysis of a given dataset when the chosen value of k is suboptimal and therefore, it becomes straightforward to select an appropriate value for it. For example, it is typically not that case that k=1 (e.g., see Figure 2B), but good fits of the multi-modal distribution can be made in most cases with k in the range 2-5. Together, the space for the analyst to reasonably explore is not too large.

5. By modeling the shape of the QC metric, rather than examining the actual values, it is difficult to evaluate the overall quality of a data sample, which is often the most important goal for QC. At an extreme, a bad sample which has few outliers may be utilized more compared to a good sample with more outliers. This seems to be an intrinsic limitation of this proposed model.

Exploring the extreme case mentioned (bad sample with few outliers versus good sample with more outliers) is covered well by the QC reports that SampleQC provides (see also Figure 4). For example, this shows a clustering of samples based on maximum mean discrepancy and can be useful to identify batch effects or issues with individual samples. We have added text to the User Guide describing this.

6. What exactly is the criteria for outliers? The description in the paper is very vague.

Thanks for this comment. The criteria for outliers was buried in the Software subsection of the Methods. We have now created a subsection titled "*Criteria for outliers*" to describe this more explicitly.

Minor comments

1. It is unclear how many QC features are considered in this model. Figure S1 indicates three metrics: counts, features, and mitochondria fractions. Is this all to be considered?

Indeed, the features to be included need to be defined by the user. In our experience, counts, features and mitochondria fractions capture the necessary features for standard single cell RNA-seq data; as mentioned in our analysis of the CITE-seq data, extra dimensions there include the number of antibody tag reads and/or the number of non-zero antibody tags observed; for single nuclei RNA-seq, the intron/exon ratio is indicative. For other modalities, other features are certainly relevant (e.g., sparseness in scATAC-seq, etc). We have now mentioned these in the Discussion (e.g., sentence fragment: "*by selecting appropriate measures (e.g., antibody tag depths for CITE-seq, sparseness for scATAC-seq, etc.)*").

2. The authors suggested the potential utility of summing up information from low quality data points. This may be true, but it should be a separate issue than QC analysis. Similarly, binarization is not an intrinsic flaw for QC analysis, as the full continuous scores can be retrieved.

We do not explicitly mention binarization and are therefore not completely confident of our interpretation of this comment, but this could mean in context of the assignment of ‘bad’ cells; indeed, as the reviewer mentions, binarization is not strictly required since the full continuous scores are available. In fact, these continuous measures are something that could be shown after a clustering analysis to explore whether entire clusters are of debatable quality. A comment (“*SampleQC summaries (e.g., P-values representing cells in low-density regions of their QC space) could be used afterwards as indicators for removal of individual clusters*”) about this is made in the Discussion.

Reviewer #3:

This manuscript describes a new quality control approach for scRNA-seq data that uses GMMs to detect outlier samples and cells to be excluded from the analysis. The authors describe the method well, justifying their modifications to the standard approaches for identifying GMMs, i.e. sample specific shift terms and using an approach to estimate means and covariance matrices that is robust to outliers. The sample-to-sample distance embedding in UMAP plots overlaid with sample metadata information is a helpful tool for assessing potentially problematic samples. Using simulated data, they show that they can detect outliers with a reduction in total counts and features, and an increase in mitochondrial proportions - typical metrics used to identify poor quality cells in single cell RNA-seq QC.

It would be helpful to have more in-depth legends for the figures, and/or more text describing the figures and how to interpret them. For example, in the analysis of real, complex data, the authors show that SampleQC detects different outlier cells compared to scater and miQC. However, while I can see why they are considered outliers based on the model, I did not find a clear biological justification for why some cells detected by SampleQC should be excluded. For example in Figure 5C,D,E, SampleQC detects some cells that have high counts and low mito and good splice ratios in the top left of 5C. What are these cells that scater and miQC retain? Do they have particular features? Perhaps further details have not been provided because the data are not published and the biology has not been described, but it would be helpful to understand what might be driving those differences.

Thank you for these comments. We have investigated and adjusted various legends in order to make them all more in-depth; these adjustments can be seen in the revised version of the manuscript in orange text.

We have investigated the QC metric distributions of the outliers identified under each method; some illustrative results (for 6 randomly selected samples) are shown below. Some conclusions can be drawn from these results, e.g., that miQC excludes all cells above a given mitochondrial threshold; that in some samples, scater excludes all cells with lower than a given library size. However, conclusions about SampleQC are by the nature of the model used more difficult to draw. Outliers are determined on the basis of relative local density, rather than globally extreme values. We would argue that this is just as valid a motivation for excluding cells as those used by scater and miQC, where we have shown that the assumptions used can result in excluding cells that are ‘good’ quality but come from celltypes with extreme QC metric values.

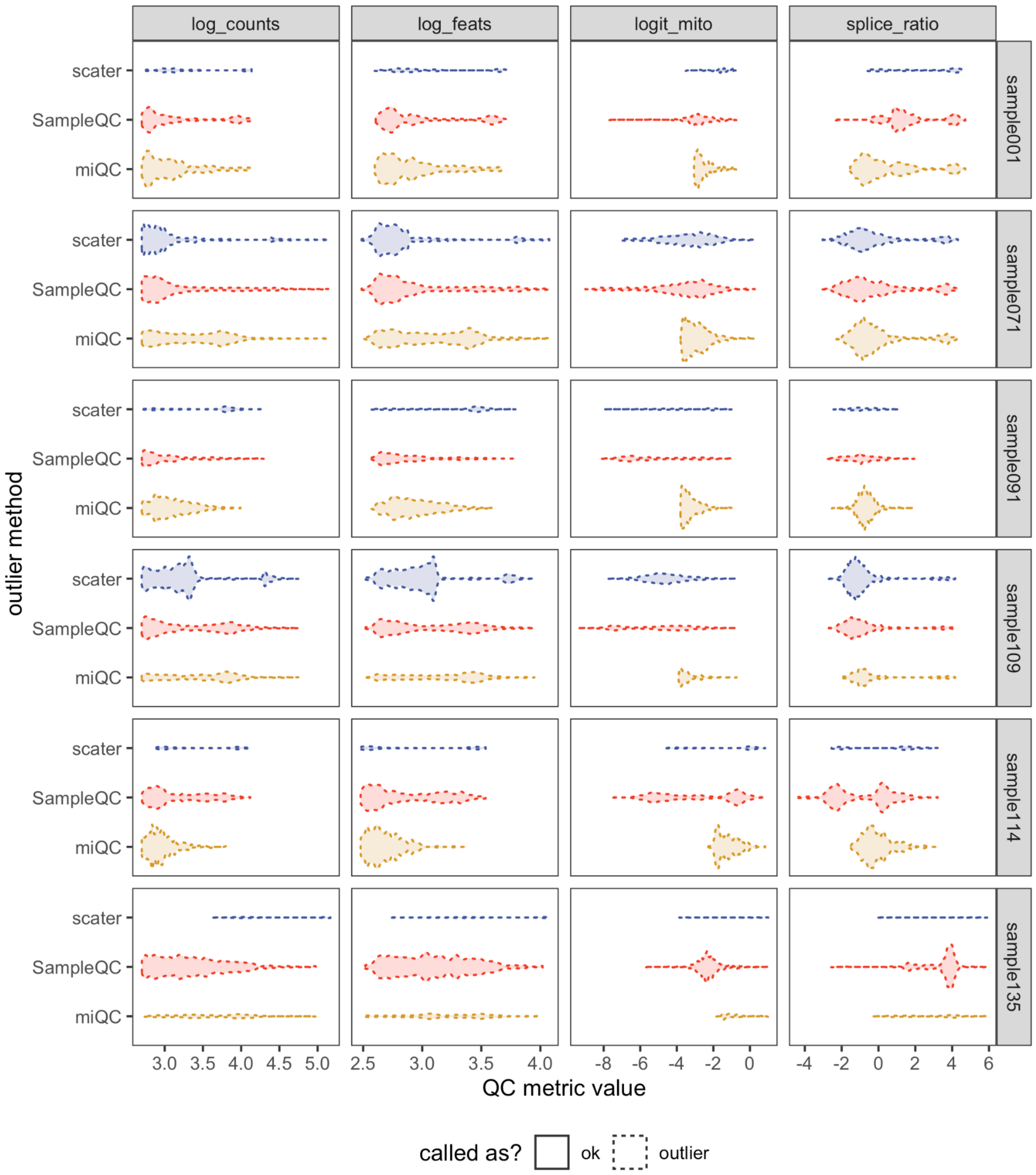

We agree that for a small number of cells, extreme values of the observed QC metrics would not be a good reason to exclude them. However, these cells are, as a result of being outliers in SampleQC, unusual in that there are very few cells with this specific **combination** of QC metrics. For example, these could be cells with QC metric values that are extreme *relative* to that cell's celltype. However, this is difficult to test, since celltype annotation occurs after exclusion of poor quality cells.

In any case, the desired benefit of SampleQC is to reduce bias in entire celltypes. We believe that the gain of rare celltypes can be worth the trade-off of losing a small number of potentially ‘good’ cells from more abundant celltypes (whose loss is unlikely to have any effect on downstream analyses).

I appreciate that the analyses have been well organized in Github using workflowr to enable reproducibility. However, I was not able to reproduce all the analyses provided in the Github link, https://github.com/wmacnair/SampleQC_paper_analyses.
- for the file qc01_prep_data.Rmd, the folder data/miqc does not exist in the repo (see https://github.com/wmacnair/SampleQC_paper_analyses/tree/main/data).
I also ran into problems running qc04_real.Rmd:
- in the "setup_helpers" chunk, I had trouble loading the file and had to explicitly set my working directory
- when running the "load_qc" chunk, I got an error "Error in colnamesInt(x, neworder, check_dups = FALSE) : argument specifying columns specify non existing column(s): cols[5]='splice_ratio'".

We thank the reviewer for pointing this out. We have adjusted the repository so that it is easier to reproduce, although we recommend using Bioconductor 3.13 / R 4.0.x in order to match the packages that we used for the analysis.

If the authors could go into greater depth regarding what the cells are that SampleQC is detecting that other methods do not, I can see this approach being of broad utility and of great interest to a broad audience of biologists and data analysts. It would also be useful if the software could accept other file formats, e.g. seurat objects, which are commonly used for scRNA-seq analysis and would allow for easier incorporation of this approach into standardized workflows.

As mentioned above, we have now given more details about the differences between what SampleQC filters and previous methods do; ultimately, we tend to exclude cells that are extreme in QC metrics within the context of similar cells. We already have a description in the github repository (see README.md) about how to use SampleQC within either a Seurat- or Bioconductor-based pipeline (ultimately, SampleQC is based on a data.frame of the QC summaries, which is fairly straightforward to extract from any current pipeline).
