# Supplementary material for: SampleQC: robust multivariate, multi-cell type, multi-sample quality control for single-cell data
Source: Genome Biol. 2023 Feb 10;24:23. doi: 10.1186/s13059-023-02859-3 (PMC9912498; doi:10.1186/s13059-023-02859-3)
Supplement: Supplementary file 4 — Additional file 4. Authors’ response to second round of review [file 13059_2023_2859_MOESM4_ESM.pdf]

You will see that while Referee 1 is now mostly satisfied with the manuscript, Referees 2 and 3 still raise concerns that their previous comments have not been fully addressed. In particular, Referee 3 is concerned that it is not clear that SampleQC is finding poor quality cells that are missed by other methods, and Referee 2 is concerned that the validation relies on the same modeling being used for simulation and model inference. I am afraid these outstanding issues are substantive and we must insist that they are addressed in the form of a further, final revision of the manuscript, before we reach a final decision on publication. In particular, we ask that you provide a better demonstration that your method is an improvement on existing methods.

We do appreciate that a lack of a gold standard dataset for assessment is a real problem. It is increasingly raised by reviewers, particularly in single cell and spatial genomics, which are relatively new fields.

Response to editor:

We agree with the sentiment of both Reviewers 2 and 3, however we do not believe that it is even possible to address these criticisms. We feel that we have discussed these aspects quite honestly and thoroughly in the Discussion, especially the limitations around the lack of ground truth for the task of QC as a whole (not just SampleQC) and that we cannot say whether an outlier is truly a ‘bad’ cell, also because there is no clear definition of bad; in particular, we have deliberately tried not to “oversell” what SampleQC is capable of. Our work simply provides additional flexibility beyond what was previously available in the community (providing a solution for some datasets where other methods did not work), but we cannot show and do not claim that our method is the best under all circumstances.

You note the problematic lack of a gold standard for QC; at least based on our own thinking and the reviewers’ comments, we believe there is therefore no further analysis that would clearly address the reviewers' concerns. For this reason (also expanded below), we do not see any value in making further substantial revisions to the manuscript (we made some further textual changes, as noted below; and, we are happy to make further changes if the editor deems them worthwhile). We nonetheless thank the editor and reviewers for this interesting and helpful discussion of an important topic.

***Reviewer #1**: In this revision Macnair & Robinson have added computational experiments and extended discussion on alternative strategies and limitations of SampleQC. This manuscript would make for a great publication.*

***Reviewer #2**: The revised paper continues to have the flaws that I pointed out in my original review, largely because they did not provide any additional evidence to support their claims. As I pointed out, a key weakness of their validation scheme is that the same model assumptions are used both for data simulation and model inference. The authors acknowledged this weakness but defended their choice by essentially saying it is very difficult to simulate data any other way. This is a striking argument and in doing so they ignored the reality that the source of variation in a real experiment can never be known in full, therefore, the role of statistical modeling is only to approximate reality rather than replace it. Model mis-specification is an important source of error that requires careful consideration, otherwise the entire procedure, however elaborate it is, simply becomes a self-fulfilling exercise.*

What the reviewer mentions here is not entirely true. Our validation scheme is not simply doing inference after sampling data directly from the model. We performed 2 additional and important extensions: (i) we sampled data with much heavier tails (as is commonly done in the statistical literature), and highlighted that our robust inference strategy handled this without problems; and, perhaps more importantly with regard to model mis-specification, (ii) we performed analysis to highlight that where the number of mixture components in the model is mis-specified, this would become apparent to a user that scans through the reports. Taken together, we think the reviewer misrepresents the validations that we had performed and at the same time acknowledges that no simulator exists to cover these aspects.

Another weakness pointed out not just by me but also by other reviewers is the lack of distinction between technical and biological variability. It is unclear why cells with outlier QC properties must be regarded as bad cells rather than healthy cells at a distinct biological state. The authors argued that their treatment would help identify rare cell types rather than making such detection model difficult, but this claim requires concrete supporting evidence.

We agree with the reviewer on this aspect. While the model does have both technical and biological variability represented, in the end, we do not know whether a cell that’s an outlier is one because of technical or biological reasons; we now added a statement to the Discussion to this effect: “*However, SampleQC (as with other QC methods) is still not able to know whether a cell listed as an outlier is one because of technical or biological reasons.*”

Two benchmarks of single cell sequencing protocols, where samples from the same mixture of cells were sequenced by different labs with different protocols (Ding et al., Mereu et al.), would allow us to explore the differences between technical and biological variability. Differences between samples from these datasets therefore represent only technical rather than biological variability. However even in this case, where outlier status depends purely on technical factors, we do not have a ground truth, and quantification of QC performance remains non-obvious.

This is therefore the crux of the additional text that we added to the Discussion in the first revision, and honestly, we do not believe it is possible for this problem (distinguishing the origin of ‘bad’ cells) to have a clear solution. In fact, this same comment could be directed at the 100s to 1000s of single cell papers published (where the large majority of Methods sections in these manuscripts have some kind of trim-outliers strategy) and also at the currently available methods for single cell QC (e.g., scater, miQC). The SampleQC framework simply offers additional flexibility for this task based on sharing information across samples.

The authors did clarify the definition of outliers in the Method section, but this is probably the only major improvement compared to the original version.

We are happy that these clarifications led to a major improvement.

Reviewer #3*: The manuscript is improved by MacNair and Robinson's clarifications regarding what cells are deemed poor quality. The point is well-taken that exclusion on the basis of relative local density may be just as valid as exclusion based on extreme QC values.*

However, I remain unconvinced that the approach is an improvement over existing methods for QC. The ovarian cancer example is intended to demonstrate that the application of minimal filtering, followed by removal of clusters with poor QC metrics after clustering, may be challenging in some types of samples. However, they do not go so far as to demonstrate how different the clustering/characterization would be with (1) filtering based on defined thresholds, (2) minimal filtering and then removal of clusters with problematic QC metrics, and (3) SampleQC analysis. It therefore remains difficult to understand which cells are being marked as poor quality by SampleQC that might not have been identified with the current approaches, and vice versa. The authors indeed state that they "do not have specific reasons that they are truly low-quality, beyond the observation that they by definition have more extreme QC metrics."

As we elaborated on in the Discussion in the revision, the metrics of success to demonstrate improvement over existing methods are really the key limitation. We have of course put considerable thought into how we can demonstrate improvement, and have been quite forthcoming in the limitations of the evidence that we did collect. The reviewer mentions looking towards “how different the clustering/characterization would be”, but this is also without a clear metric of success given the absence of ground truth. The reviewer does acknowledge that local density is as valid a filtering criterion as any.

The reviewer requires that our method is an improvement over existing methods for QC. We believe we have shown that our method is an improvement for at least some datasets (we think for an important class of challenging datasets). Requiring a method to be better than others in *all circumstances* is both unrealistic and unfair: different datasets have different characteristics and therefore require different approaches. For example, a recent comprehensive benchmark of data integration methods for single cell discusses the trade-off between removing differences between batches and conserving true biological variation; the ideal balance between these depends on the user’s needs, and the benchmark suggests a range of methods all of which are

potentially optimal depending on the chosen balance (Luecken *et al.*). We see our method as one in a range of methods that can be optimal, and it offers considerable additional flexibility to existing approaches, especially for the now-common multi-sample situation.
