# Supplementary material for: SampleQC: robust multivariate, multi-cell type, multi-sample quality control for single-cell data
Source: Genome Biol. 2023 Feb 10;24:23. doi: 10.1186/s13059-023-02859-3 (PMC9912498; doi:10.1186/s13059-023-02859-3)
Supplement: Supplementary file 2 — Additional file 2. Reviewer reports. [file 13059_2023_2859_MOESM2_ESM.docx]

**1^st^ round**

**Reviewer 1**

Review

Macnair & Robinson propose a version of a Gaussian mixture model to be applied to summarized QC metrics in scRNA-seq data to identify outliers corresponding to 'bad' cells.

The model, which uses robust inference to fit in the presence of outliers as well as including batch-specific mean shifts, is beautifully designed with a very nice corresponding R package that could find use in many fields beyond scRNA-seq.

The use of logistic transformation of the mitochondrial proportion is a great idea; the logistic transformation of fractions is very underutilized in the field.

The simulation strategy outlined by authors is comprehensive and appropriate. Data is simulated according to the specification of the model proposed by the authors. They demonstrate clearly that the model will successfully be able to recover the simulation conditions better than alternatives. While the results are positive and encouraging (mixture models are hard to get fitting correctly even for data simulated directly from the models!), it only demonstrates inference is possible rather than truly identifying 'bad' cells.

As opposed to many modeling + single cell papers, here the authors have properly quantified the performance of theirs and alternative metrics with precision-recall curves. It would be great if these results were presented in Figure 2, which is more useful than the example scatter plots currently presented which doesn't quantify the performance and instead relies on mental pattern matching by a reader.

Cells are labeled as 'poor quality' or 'bad' if they are far into the tails of the Gaussian mixture components. It is not clear why these cells would be of poor quality as opposed to following biological variation with wider distribution than can be represented by a multivariate normal. Often real world data need to be modeled with t-distribution rather than Gaussian distributions to properly mimic the observed data.

Relatedly, the assumption of the cell QC GMM is that large clusters correspond to useful cells while outliers correspond to 'bad' cells. However, in practice it is common to find a distinct cluster between QC metrics which is enriched for various markers of cell death or degradation.

It is a reasonable theory that different biological cell types will produce different clusters in 'QC space'. But for none of the datasets is it demonstrated that different cell types indeed have different QC distributions. For the sake of a reader it would be appropriate to relate examples of different kinds of cell types that can have different QC values and why.

Another assumption is that the majority of cells will be 'good'/useful. However, for some experimental systems with particularly fragile cells a majority will be 'bad'/dead. Since modern platforms can capture tens of thousands of cells per sample it tends to not be an issue since you can still capture hundreds of useful cells. The authors should provide guidance regarding these settings.

The authors write that excluding cells with more than 5%/10% mitochondrial reads is a common approach to QC. It would be good if the authors demonstrated with a few recent example publications to illustrate the prevalence of this practice. The authors do have a reference to a review recommending this, but this does not indicate to a reader that this is a widely used practice in the field.

It is stated that the current industry standards for QC are 'scater' and 'Seurat'. It would be good to provide examples of the use of this strategy. Reading papers from the field, it seems far more common to make arbitrary cutoffs on a couple of custom metrics in each paper.

The authors mentions that applying QC filtering to each sample individually introduces bias, but this is not investigated or presented in this paper.

Towards the end of the paper the authors state that jointly analyzing all samples increases sensitivity by borrowing strength across samples. There is however no discussion of comparison of performance of analyzing the samples individually as opposed to jointly.

A large issue is how this method fits into an analysis workflow. The central claim is that different cell types of useful cells will produce different densities of QC metrics. For a practitioner to make use of data with multiple cell types, these need to be annotated for further analysis. But in this setting 'bad' cells would form their own clusters or fail to be annotated in more supervised settings. Since the work of clustering cells and labeling still needs to be done, it would seem easier to identify 'bad' cells at that step.

It would be appropriate to discuss which QC metrics to use, and how they relate to potential failure modes in the scRNA-seq process. The authors do discuss this for the intronic vs exonic ratio of reads for snRNA-seq and offer a plausible theory (albeit speculative, which could use a qualifier to indicate this).

Upon seeing the facet_grid in Figure S2 it is not obvious what the vertical axis represent. It appears to be individual samples; it would be good if the authors used the 'labeller = "label_both"' option to make this clear.

In summary, the largest issues are: 1) It is not clear whether cells labelled as 'bad' here are 'bad' as opposed to one of the QC clusters being 'bad'. 2) Whether performing this labeling would be useful in the first case since in the clustering step of analysis researchers would identify bad cells anyway.

**Reviewer 2**

Macnair and colleagues develop SampleQC for single-cell RNA-seq data quality control (QC). They do not propose new QC criteria, but an approach to combine multiple existing QC criteria to better identify poor quality cells. To this end, they propose a multivariate Gaussian mixture model, and treat outliers from the fitted model as low quality data points. They evaluate their method using simulated data, which is based on their proposed model, then apply their model to analyze a publicly available real single nuclei RNAseq dataset from human brain.

The proposed method is incremental compared to existing approaches. There are a few merits of the proposed model. Perhaps the main advantage of this approach is that it does not require a predefined threshold for certain QC metrics, such as UMI counts and mitochondria fractions, but the model requires additional parameters that are not easily identifiable from data, e.g. QC celltype numbers and threshold for outliers. The model is not well validated. In addition, there are a number of significant weaknesses as detailed below.

Major comments:

1. The proposed method lacks rigorous validation. Quantitative evaluation is done only for a specific simulated dataset, which is generated based on the assumption their statistical model is correct. This is clearly a circular argument, and the relevance for real data application is unclear. There are a number of published tools for single-cell RNAseq simulations. The authors should consider one of multiple of these tools in order to obtain a more objective evaluation of their method. Also, simulated data has important intrinsic limitations which need to be recognized. To avoid undue optimism in real applications, the authors should also carefully evaluate the performance of their method based on real data. I recognize it can be difficult to determine true error rates for real data, but alternative strategies can be used such as robustness and consistency with literature findings, as commonly done in similar studies.

2. A key challenge in QC analysis is to distinguish good from bad data points while considering the underlying biological variability. As noted by the authors, variation in metrics such as UMI counts and mitochondrial fractions may be due to intrinsic biological variation. However, this issue can only be addressed by carefully modeling the sources of biological and technical variations. Instead, a key limitation of sampleQC is that it does not attempt to distinguish biological and technical components. As a result, it is hard to interpret the results. An example is that rare cell types may appear outliers that would be excluded from downstream analysis. Similarly, the term 'QC celltype' is confusing and hard to interpret.

3. The benefit of using this proposed QC metric in downstream analyses is unclear. For example, does it help improve the accuracy of cell-type identification, trajectory analysis, etc?

4. A key parameter in sampleQC is the number k for GMM. It is unclear how this parameter is chosen. The authors recommend the users to inspect a few plots generated by the program to make their own choice, but this is very difficult without a guiding principle. With this key parameter unsettled, it is also very difficult to evaluate the performance of the model for real data.

5. By modeling the shape of the QC metric, rather than examining the actual values, it is difficult to evaluate the overall quality of a data sample, which is often the most important goal for QC. At an extreme, a bad sample which has few outliers may be utilized more compared to a good sample with more outliers. This seems to be an intrinsic limitation of this proposed model.

6. What exactly is the criteria for outliers? The description in the paper is very vague.

Minor comments

1. It is unclear how many QC features are considered in this model. Figure S1 indicates three metrics: counts, features, and mitochondria fractions. Is this all to be considered?

2. The authors suggested the potential utility of summing up information from low quality data points. This may be true, but it should be a separate issue than QC analysis. Similarly, binarization is not an intrinsic flaw for QC analysis, as the full continuous scores can be retrieved.


===
Please consider the following questions:
- Are the methods appropriate to the aims of the study, are they well described, and are necessary controls included?
- Are the conclusions adequately supported by the data shown?
- Are sufficient details provided to allow replication and comparison with related analyses that may have been performed?
- Is the method likely to be of broad utility? Is any software component easy to install and use? Please indicate briefly the novel features and/or advantages of the method, and/or please reference the relevant publications and which methods, if any, it should be compared with.
- Is the paper of broad interest to others in the field, or of outstanding interest to a broad audience of biologists?

**Reviewer 3**

This manuscript describes a new quality control approach for scRNA-seq data that uses GMMs to detect outlier samples and cells to be excluded from the analysis. The authors describe the method well, justifying their modifications to the standard approaches for identifying GMMs, i.e. sample specific shift terms and using an approach to estimate means and covariance matrices that is robust to outliers. The sample-to-sample distance embedding in UMAP plots overlaid with sample metadata information is a helpful tool for assessing potentially problematic samples. Using simulated data, they show that they can detect outliers with a reduction in total counts and features, and an increase in mitochondrial proportions - typical metrics used to identify poor quality cells in single cell RNA-seq QC.

It would be helpful to have more in-depth legends for the figures, and/or more text describing the figures and how to interpret them. For example, in the analysis of real, complex data, the authors show that SampleQC detects different outlier cells compared to scater and miQC. However, while I can see why they are considered outliers based on the model, I did not find a clear biological justification for why some cells detected by SampleQC should be excluded. For example in Figure 5C,D,E, SampleQC detects some cells that have high counts and low mito and good splice ratios in the top left of 5C. What are these cells that scater and miQC retain? Do they have particular features? Perhaps further details have not been provided because the data are not published and the biology has not been described, but it would be helpful to understand what might be driving those differences.

I appreciate that the analyses have been well organized in Github using workflowr to enable reproducibility. However, I was not able to reproduce all the analyses provided in the Github link, https://github.com/wmacnair/SampleQC_paper_analyses.
- for the file qc01_prep_data.Rmd, the folder data/miqc does not exist in the repo (see https://github.com/wmacnair/SampleQC_paper_analyses/tree/main/data).
I also ran into problems running qc04_real.Rmd:
- in the "setup_helpers" chunk, I had trouble loading the file and had to explicitly set my working directory
- when running the "load_qc" chunk, I got an error "Error in colnamesInt(x, neworder, check_dups = FALSE) : argument specifying columns specify non existing column(s): cols[5]='splice_ratio'".

If the authors could go into greater depth regarding what the cells are that SampleQC is detecting that other methods do not, I can see this approach being of broad utility and of great interest to a broad audience of biologists and data analysts. It would also be useful if the software could accept other file formats, e.g. seurat objects, which are commonly used for scRNA-seq analysis and would allow for easier incorporation of this approach into standardized workflows.

**2^nd^ round**

**Reviewer 1**

In this revision Macnair & Robinson have added computational experiments and extended discussion on alternative strategies and limitations of SampleQC. This manuscript would make for a great publication.

**Reviewer 2**

The revised paper continues to have the flaws that I pointed out in my original review, largely because they did not provide any additional evidence to support their claims. As I pointed out, a key weakness of their validation scheme is that the same model assumptions are used both for data simulation and model inference. The authors acknowledged this weakness but defended their choice by essentially saying it is very difficult to simulate data any other way. This is a striking argument and in doing so they ignored the reality that the source of variation in a real experiment can never be known in full, therefore, the role of statistical modeling is only to approximate reality rather than replace it. Model mis-specification is an important source of error that requires careful consideration, otherwise the entire procedure, however elaborate it is, simply becomes a self-fulfilling exercise.

Another weakness pointed out not just by me but also by other reviewers is the lack of distinction between technical and biological variability. It is unclear why cells with outlier QC properties must be regarded as bad cells rather than healthy cells at a distinct biological state. The authors argued that their treatment would help identify rare cell types rather than making such detection model difficult, but this claim requires concrete supporting evidence.

The authors did clarify the definition of outliers in the Method section, but this is probably the only major improvement compared to the original version.

**Reviewer 3**

The manuscript is improved by MacNair and Robinson's clarifications regarding what cells are deemed poor quality. The point is well-taken that exclusion on the basis of relative local density may be just as valid as exclusion based on extreme QC values.

However, I remain unconvinced that the approach is an improvement over existing methods for QC. The ovarian cancer example is intended to demonstrate that the application of minimal filtering, followed by removal of clusters with poor QC metrics after clustering, may be challenging in some types of samples. However, they do not go so far as to demonstrate how different the clustering/characterization would be with (1) filtering based on defined thresholds, (2) minimal filtering and then removal of clusters with problematic QC metrics, and (3) SampleQC analysis. It therefore remains difficult to understand which cells are being marked as poor quality by SampleQC that might not have been identified with the current approaches, and vice versa. The authors indeed state that they "do not have specific reasons that they are truly low-quality, beyond the observation that they by definition have more extreme QC metrics."
